# A previously unidentified circRNA inhibits virus replication by regulating the miR-24-3p/KEAP1 axis

**DOI:** 10.1371/journal.ppat.1012712

**Published:** 2024-12-17

**Authors:** Haimin Li, Liuyang Du, Juan Li, Yanming Huang, Chenhe Lu, Tingjuan Deng, Yan Yan, Yulan Jin, Wei Wu, Jinyan Gu, Jiyong Zhou

**Affiliations:** 1 MOA Key Laboratory of Animal Virology, Zhejiang University Center for Veterinary Sciences, Hangzhou, China; 2 State Key Laboratory for Diagnosis and Treatment of Infectious Diseases, First Affiliated Hospital, Zhejiang University, Hangzhou, China; Vanderbilt University Medical Center, UNITED STATES OF AMERICA

## Abstract

Circular RNAs (circRNAs) exert diverse biological functions in different processes. However, the role of circRNAs during virus infection is mostly unknown. Herein, we explored the characteristics of host circRNAs using alphaherpesvirus pseudorabies virus (PRV) as a model. PRV infection upregulated the expression of circRNA circ29164, which does not encode a protein. RNA pulldown assays identified that circ29164 interacts with the microRNA ssc-miRNA-24-3p. Further analysis indicated that ssc-miR-24-3p targets the mRNA encoding kelch-like ECH-associated protein 1 (KEAP1), and circ29164 competitively binds to ssc-miR-24-3p to prevent it binding to *Keap1*. Apoptosis detection demonstrated that circ29164 or *Keap1* overexpression, but not knockdown, induced caspase 3 activity and the release of cytochrome C from mitochondria, and inhibited PRV replication. Taken together, these data identified a previously undiscovered circRNA, circ29164, which inhibits PRV replication by competitively binding to ssc-24-3p to maintain KEAP1 levels.

## Author summary

As an important epidemic affecting pig farming, Pseudorabies (PR) outbreaks in China since 2011, caused by variant Pseudorabies virus (PRV) (genotype II) in Bartha-K61-vaccinated pigs, have resulted in considerable economic losses. In addition, studies have proven that the variant PRV has been transmitted to humans and could be isolated from human patients with ophthalmitis or encephalitis. Herein, we found that a previously unidentified circular RNA (circ29164) is induced by and inhibits PRV infection. Specifically, upregulated circ29164 sponges the microRNA ssc-miR-24-3p, which targets the mRNA encoding kelch-like ECH-associated protein 1 (KEAP1), thereby maintaining KEAP1 levels. This results in intrinsic cell apoptosis triggered by the release of cytochrome C in mitochondria, thereby inhibiting virus proliferation. This phenomenon was observed in other viruses, such as influenza virus and porcine delta coronavirus. Our data enriched the regulatory mechanism of circRNAs in virus infection and provides a new potential antiviral target for the prevention and treatment of viral diseases.

## Introduction

Pseudorabies virus (PRV), with a genomic double-stranded DNA of about 140 kb, is a member of the *Alphaherpesvirus* subfamily, which causes typical neurological symptoms in a variety of susceptible animals [1]. PRV can also establish latent infection in the nervous system without any clinical signs [2] and is activated when exposed to unfavorable external factors, resulting in recurrent infection [3]. PRV can be used as a tool in neuronal circuit tracing [4], gene therapy [5] and recombinant vaccine vector development [6,7] because of its neuropreference and foreign gene tolerance. PRV is extremely infectious, causing a systemic viral disease with severe clinical symptoms, such as itching and opisthotonos in piglets, as well as abortions and stillbirths in sows. Since the end of 2011, a novel PRV infection, identified as PRV type II (PRV-II), has spread widely in pig herds in mainland China, even in those vaccinated with PRV vaccine (Bartha-K61 strain), causing 100% mortality in experimental swine infection [8]. Research showed that PRV-II and the Bartha-K61 strain induce different cellular responses [9]. Unexpectedly, PRV-II has repeatedly been transmitted to humans in China and has been isolated from human patients with ophthalmitis or encephalitis [10,11]. Although currently incapable of sustained human-to-human transmission, PRV undoubtedly poses a threat to public health. Hence, preparing for such a threat is a global priority.

Noncoding RNAs play an important role in the virus life cycle, and their identification can provide important clues to develop strategies to control viral diseases. Circular RNAs (circRNAs) are novel endogenous noncoding RNA molecules that are covalently linked end-to-end, making them more stable than linear RNA. They are widely found in a variety of organisms, comprising different variants with different splicing sites [12]. CircRNAs can be regulated by RNA polymerase, cis or trans regulatory factors, and circRNA turnover [13]. Increasing evidence indicates that circRNAs act as microRNA (miRNA) sponges [14], affect the splicing of their linear cognates and the gene expression of linear RNAs, or can be used as disease-associated diagnostic biomarkers [15]. CircRNAs are multifunctional, being used in cancer diagnosis and treatment [16], as a kind of vaccine [17,18], in the regulation of kinases [19], and as antiviral molecules [20]. Recently, we detected the landscape of circRNA expression during virus infection, and noted that many circRNAs were differently expressed [21]. However, the functions of circRNAs during PRV infection have not been reported.

Apoptosis is a type of programmed cell death that is important to organismal development and resistance to pathogen invasion. Viral infection stimulates host cells to initiate apoptosis to inhibit virus replication. Apoptosis can be triggered by both extrinsic and intrinsic pathways, and is characterized by chromatin condensation, nuclear fragmentation, and apoptotic body formation [22]. Mitochondrial membrane permeabilization is the hallmark of intrinsic apoptosis [23]. According to the mitochondrial intermembrane space proteins that are released into the cytosol, such as apoptosis-inducing factor (AIF) [24], endonuclease G (EndoG) [25], and cytochrome C (cyt C) [26], mitochondrial membrane permeabilization-induced apoptosis can be divided into caspase-dependent or caspase-independent pathways. AIF and EndoG induced caspase-independent apoptosis by translocating to the nucleus and causing chromatin condensation and DNA fragmentation [27]. By contrast, cyt C can form an apoptosome by binding to apoptotic protease activating factor, followed by the recruitment and proteolytic maturation of procaspase-9, resulting in the activation of caspase-9 (casp-9) and downstream executioner caspases to induce apoptosis [28]. However, whether circRNAs are involved in apoptosis mostly unknown.

In this study, we identified a novel noncoding circRNA, circ29164, which was significantly upregulated after PRV infection. Overexpression of circ29164 induced apoptosis of porcine kidney (PK-15) cells and inhibited PRV replication. Knockdown of circ29164 promoted PRV replication. Our data further revealed that circ29164 competitively binds to miRNA ssc-miR-24-3p, thereby promoting the expression of *Keap1*. This results in intrinsic apoptosis by triggering the release of mitochondrial cyt C, thus inhibiting PRV replication.

## Results

### Identification and characterization of circRNA circ29164

**Fig 1:**
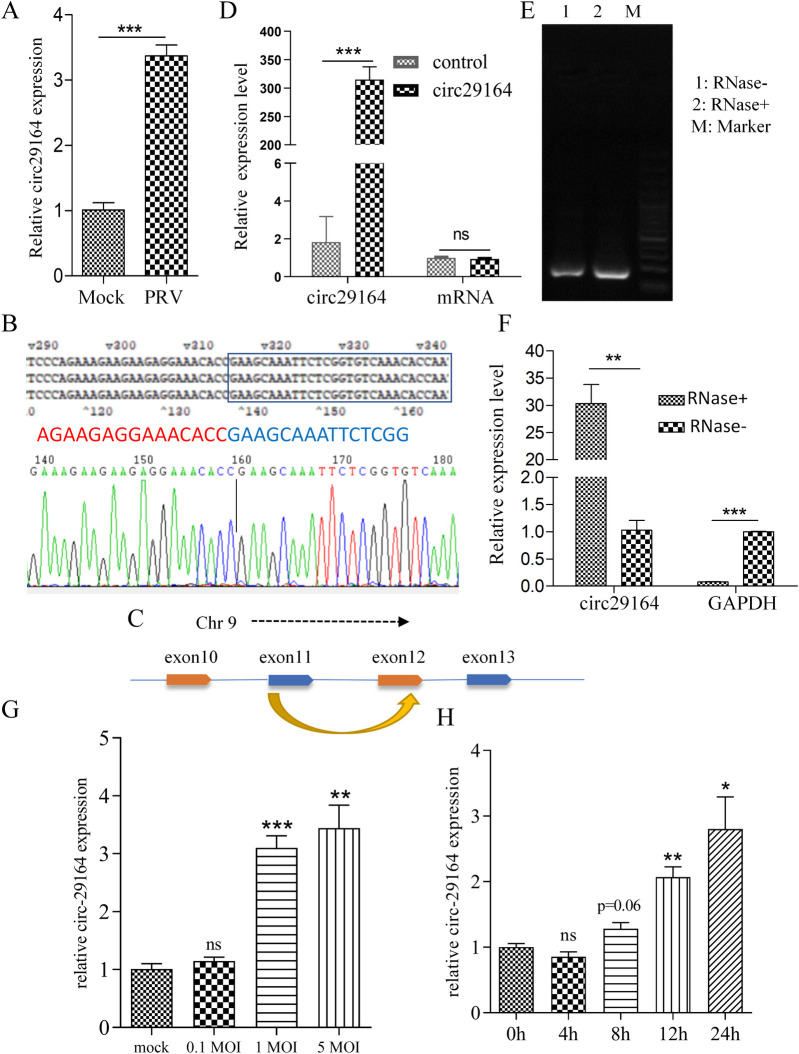
Identification of circ29164 in alphaherpesvirus-infected PK-15 cells. (A) Verification of differentially expressed circ29164 in alphaherpesvirus PRV-infected and uninfected PK-15 cells using RT-qPCR. (B) The back-splicing junction sequence of circ29164 transcript was determined. (C) Diagram of the genomic location of circ29164. (D) Verification of the circ29164 overexpressing vector by RT-qPCR. (E) RT-PCR and (F) RT-qPCR assays showing the resistance of circ29164 transcript isoforms to RNase R digestion. (G and H) Expression levels of circ29164 in PK-15 cells infected with different doses of PRV (G) and at different times (H). Values are the means ± SDs. *, *p* < 0.05; **, *p* < 0.01; ***, *p* < 0.001.

Considering the previously determined expression profiles of circRNA in PRV-infected cells [21], quantitative real-time reverse transcription PCR (RT-qPCR) was used to show that circ29164 was significantly upregulated after PRV infection of 5 MOI for 11 h (Fig 1A). Sequencing of the back-splicing junction of circ29164 showed that it was consistent with the RNA sequencing data (Fig 1B) and circ29164 was 630 nt in length. Sequence alignment to porcine genome showed that circ29164 consists of exons 11 and 12 of chromosome 9 (Fig 1C). Subsequently, a eukaryotic expression plasmid encoding circ29164 was constructed with flanking sequences that might promote its circularization, which was transfected into PK-15 cells. Subsequent RT-qPCR assays showed that the circRNA was correctly cyclized and overexpressed, whereas there was no significant difference in the linear mRNA levels (Fig 1D), indicating that circ29164 is a circRNA. To detect whether circ29164 is resistant to RNase, total cellular RNA was digested using RNase R and subjected to RT-PCR and RT-qPCR assays. The data indicated that circ29164 was enriched by RT-PCR and RT-qPCR (Fig 1E–1F), demonstrating that it is tolerant to RNase R digestion. Subsequently, we further explored the expression of circ29164 at different doses of PRV (infected for 18 h) and different times (0.5 MOI) after infection. The results showed that the upregulation of circ29164 in PRV infected samples was dose-dependent and time-dependent (Fig 1G–1H). Collectively, these data demonstrated that circ29164 is a circRNA regulated by virus infection.

### Circ29164 does not encode a protein

**Fig 2:**
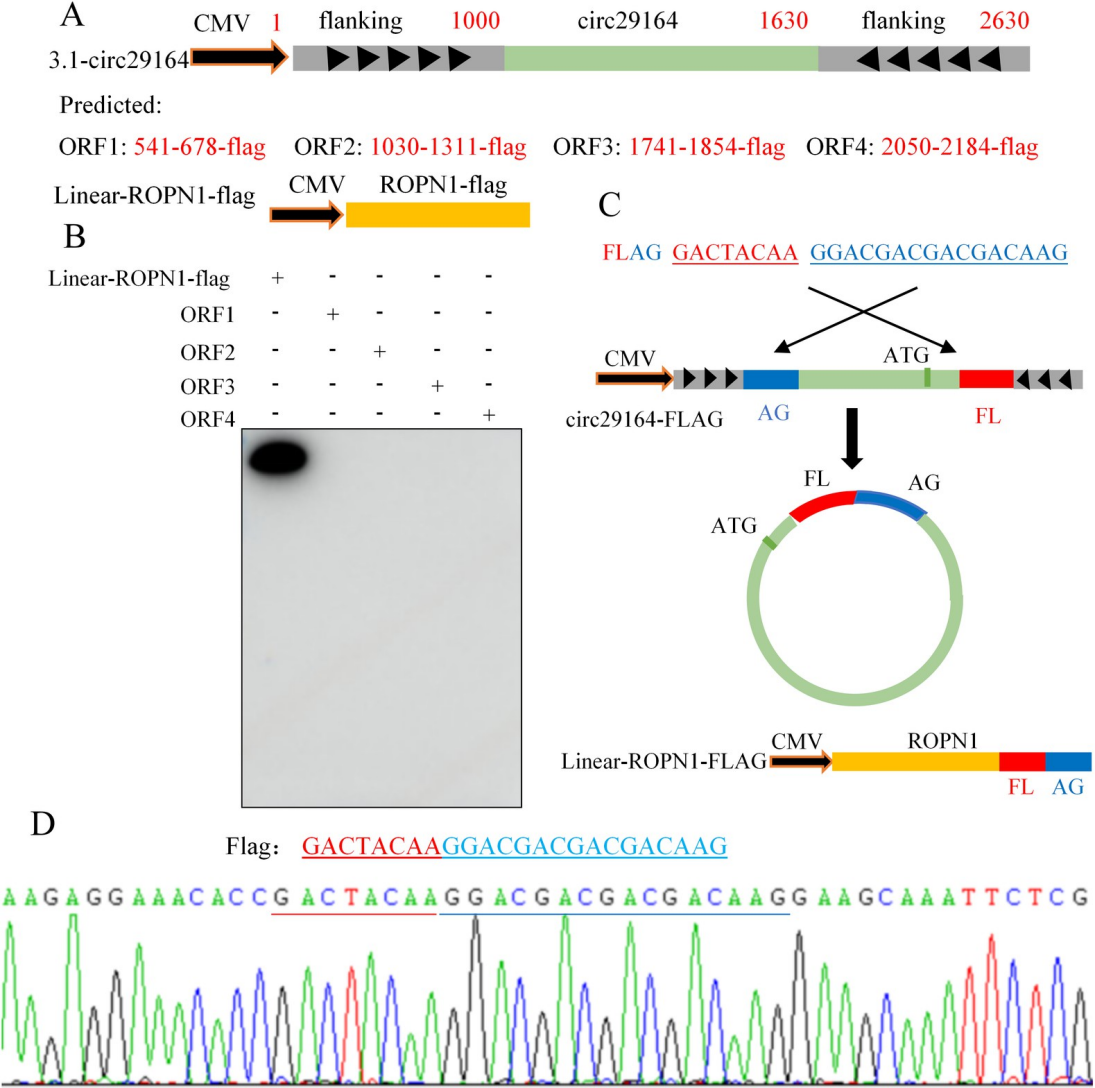
Detection of the coding ability of circ29164. (A) Diagram of the structure of the predicted open reading frames (ORFs) in the overexpressing plasmid of circ29164 and linear ROPN1-FLAG. (B) Detection of the products from the predicted ORFs and control plasmid using anti-FLAG antibodies. (C) Diagram of the structure of the predicted ORF spanning the junction of circ29164. (D) Detection of the exact FLAG sequence spanning the junction of circ29164.

A previous study showed that some circRNAs might function by encoding a protein product via an internal open reading frame (ORF) [29]. To assess the coding ability of circ29164 over-expressing vector (including the flanking sequence), the possible linear ORFs were predicted, and their eukaryotic expression plasmids were constructed, as shown in Fig 2A. A FLAG tag was added to the end of each predicted ORF and the translation of FLAG was then detected. The plasmid linear ROPN1-flag was used as the control. The results showed that none of the four predicted ORFs encoded a protein product (Fig 2B). In addition, there was a potential spanning junction ORF that might encode a 39 aa product. Subsequently, the FLAG sequence was divided into two segments and inserted into both ends of circ29164 (without frameshift for the predicted ORF), along with the flanking sequence (Fig 2C). After transfection, the complete FLAG sequence was detected, indicating that the recombinant plasmid was correctly cyclized (Fig 2D); however, no protein product was detected. These data demonstrated that circ29164 is a circRNA without encoding a protein.

### Circ29164 inhibits viral replication by promoting apoptosis

**Fig 3:**
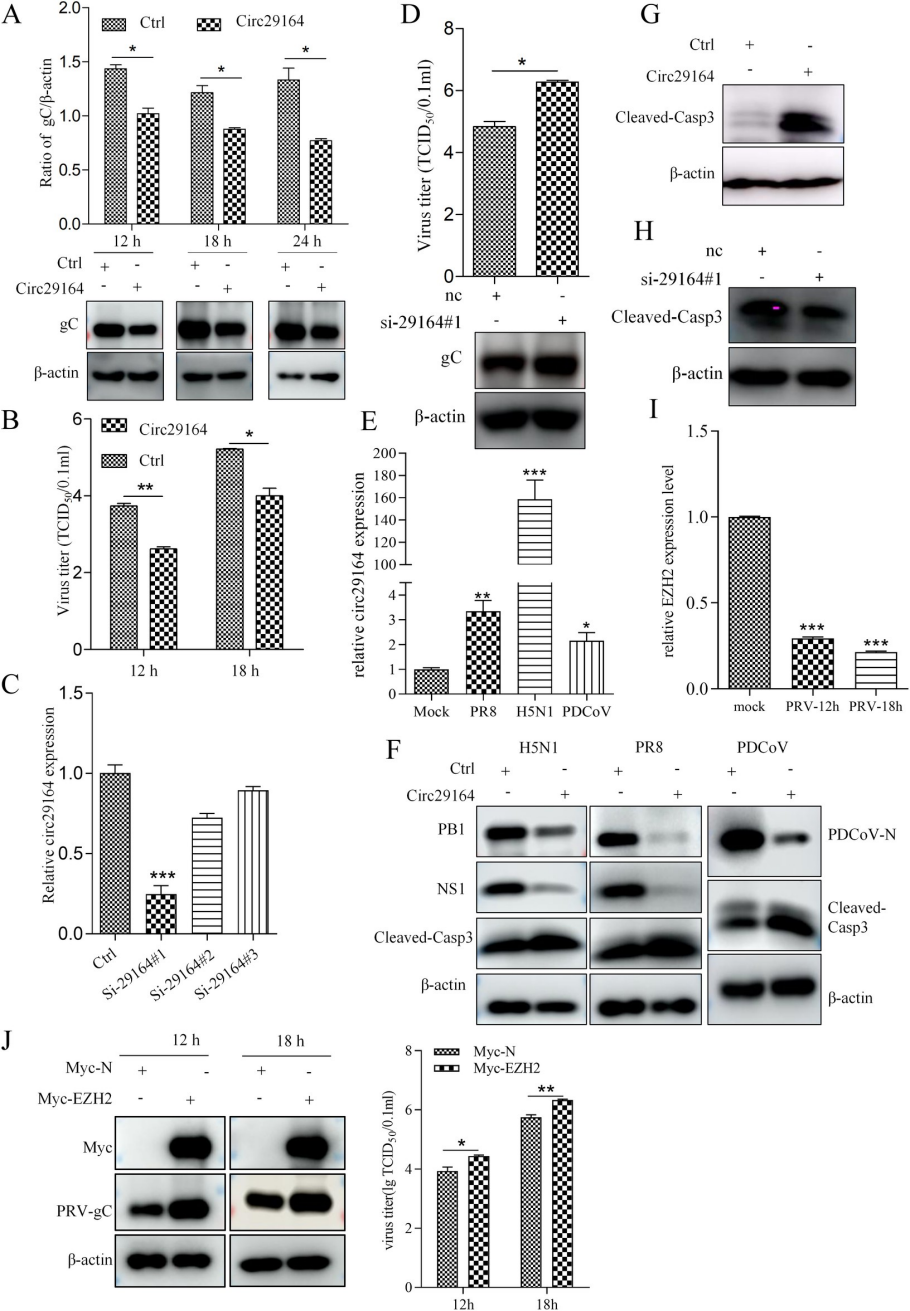
Circ29164 inhibits virus replication by promoting apoptosis. (A and B) Overexpression of circ29164 downregulated the viral protein gC expression (A) and the virus titer (B) of PRV. Circ29164 overexpressing PK-15 cells were infected with PRV at an MOI of 0.5 for the indicated times, cells and supernatant were measured for viral protein expression and the virus titer, respectively. (C) Screening of an siRNA targeting circ29164. (D) Knockdown of circ29164 promoted the viral protein gC and virus titer of PRV-DX. Cells transfected with si-29164#1 for 48 h to knock down circ29164 were infected with PRV for 12h. (E) Differentially expression of circ29164 in AIV or PDCoV-infected and uninfected PK-15 cells assessed using RT-qPCR. (F) Circ29164 overexpressing PK-15 cells were infected with PDCoV or different subtypes of influenza virus for 24 h at the MOI of 0.5, followed by detection of cell apoptosis and viral proteins. (G) Overexpression of circ29164 induced cell apoptosis. (H) Knockdown of circ29164 inhibited cell apoptosis. (I) The expression of parent mRNA after PRV infection. PK15 cells were infected with PRV of 0.5 MOI for indicated time and the mRNA was detected by RT-qPCR. (J) The effect of EZH2 on PRV replication. PK15 cells were overexpressed with EZH2 for 24 h followed by PRV infection of 0.5 MOI for indicated time. Values are the means ± SDs. *, *p* < 0.05; **, *p* < 0.01; ***, *p* < 0.001.

To explore the function of circ29164 during virus infection, PK-15 cells were transfected with pcDNA3.1-circ29164 for 24 h and then infected with PRV for the indicated times. The cell lysates were acquired for immunoblotting, and the supernatants were submitted for TCID_50_ (50% tissue culture infectious dose) assays. As shown in Fig 3A–3B, both the accumulation of viral protein gC and viral titers were lower in the circ29164 overexpressing cells than in the control cells. However, when the expression of circ29164 was interfered with using small interfering RNAs (siRNAs) (Fig 3C), the replication of PRV was enhanced (Fig 3D), indicating that circ29164 inhibits PRV replication. Similarly, porcine delta coronavirus (PDCoV) and different subtypes of Avian influenza virus (AIV) promoted circ29164 expression and were also inhibited by overexpression of circ29164 (Fig 3E–3F). Next, we detected the effect of circ29164 on PK-15 cells. The results revealed that overexpression of circ29164 induced upregulation of cleaved-casp-3 (Fig 3G), and knockdown of circ29164 downregulated the level of cleaved-casp-3 (Fig 3H), suggesting that circ29164 expression activates cellular apoptosis. Additionally, to comprehensive understand the mutual regulation between virus and circ29164, the change of the parent mRNA of circ29164 (EZH2) after PRV infection and its effect on viral replication were assessed. Results showed that the expression of EZH2 was significantly down-regulated after PRV infection (Fig 3I) and that the expression of EZH2 significantly enhanced the replication of PRV (Fig 3J). Therefore, the results indicated that circ29164 inhibits virus replication by activating cellular apoptosis, suggesting that circ29164 is a host antiviral factor.

### Circ29164 activates endogenous apoptosis by inducing cytochrome C release

**Fig 4:**
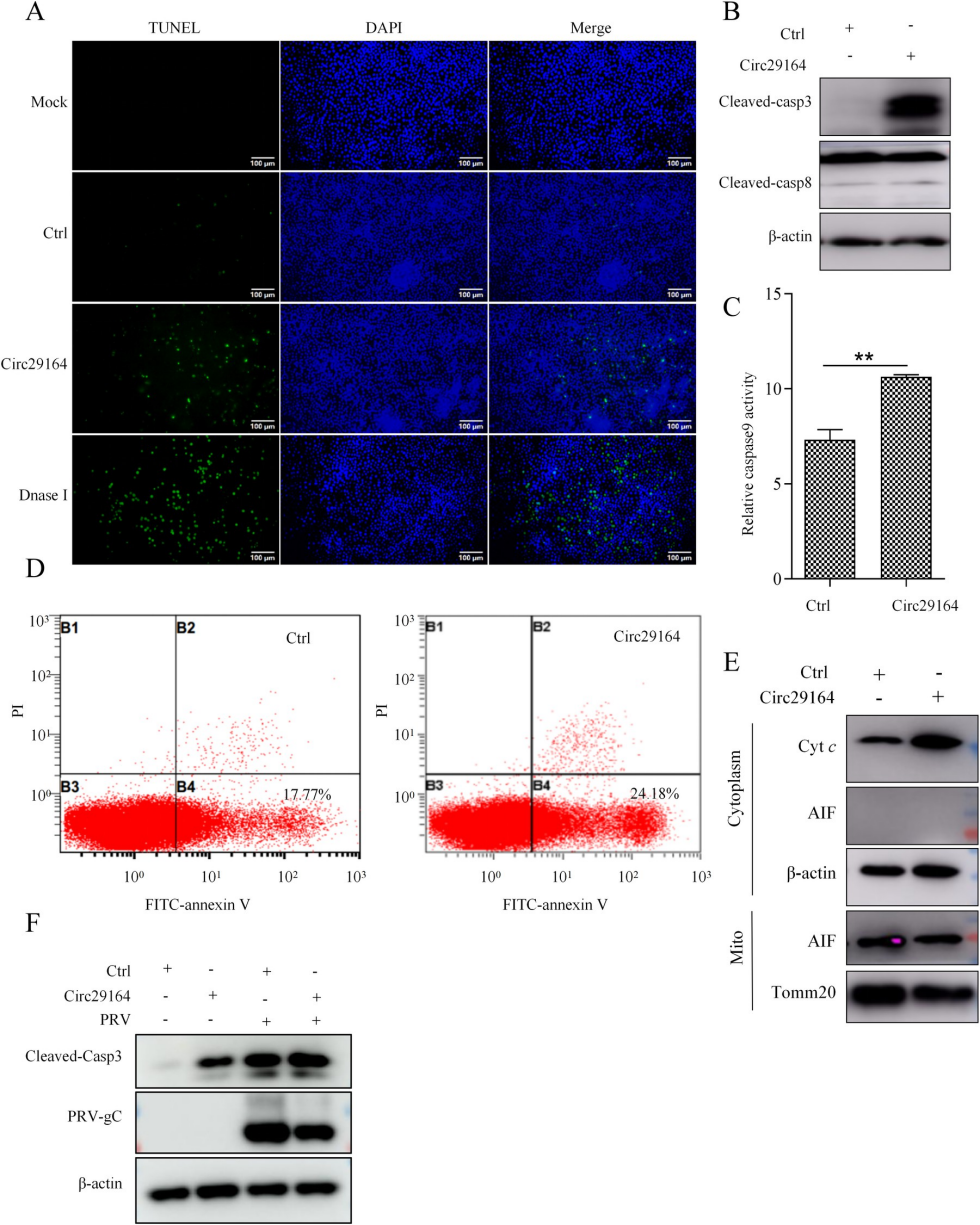
Circ29164 induces caspase-dependent mitochondrial apoptosis. (A) The effect of circ29164 on apoptosis by using TUNEL assay. (B and C) Detection of the activity of caspase 3, caspase 8, and caspase 9. Circ29164 was overexpressed in PK-15 cells for 36 h before the cleaved-casp-3, cleaved-casp-8, activation of caspase 9 were detected. (D) Circ29164-overexpressing PK15 cells were stained with PI and Annexin V and then detected using flow cytometry. (E) The detection of released cytochrome c and AIF from mitochondria. Circ29164-overexpressing PK15 cells were subjected to mitochondrial extraction and then the release of cytochrome c and AIF were detected. (F) The effect of circ29164 on apoptosis of PRV infected PK15 cells. Values are the means ± SDs. *, *p* < 0.05; **, *p* < 0.01; ***, *p* < 0.001.

To further confirm the effect of circ29164 on apoptosis, a terminal deoxynucleotidyltransferase-mediated dUTP-biotin nick end labeling (TUNEL) assay was used to detect intracellular fragmented DNA when PK15 cells were transfected with circ29164 for 36 h. As shown in Fig 4A, more TUNEL-positive cells were observed in circ29164 expressing cells compared with contral. Apoptosis can be triggered by either the extrinsic pathway or the intrinsic pathway [30]. To further define the pathway of circ29164-triggered apoptosis, we detected the activity of caspase 3, 8, and caspase 9 in circ29164-overexpressing PK-15 cells. The results showed that the activities of caspase 3 and caspase 9, but not caspase 8, were significantly induced (Fig 4B–4C), indicating the activation of intrinsic apoptosis. To investigate the period of apoptosis induced by circ29164, cells were incubated with propidium iodide (PI) and Annexin V. Flow cytometry revealed that the Annexin V signal, rather than the PI signal, was upregulated in circ29164-overexpressing cells, demonstrating the triggering of the early apoptosis (Fig 4D). Subsequently, we analyzed whether the induction of apoptosis was associated with the release of cyt C. The results showed that overexpression of circ29164 induced the robust release of cyt C, but not AIF (Fig 4E). These results showed that circ29164 induces apoptosis via the caspase-dependent mitochondrial pathway. Considering the apoptosis induction of PRV, we investigated the role of circ29164 in regulation of apoptosis during virus infection. PK15 cells were infected with PRV of 0.5 MOI for 12 h after circ29164 was overexpressed for 24 h. Results showed that circ29164 can aggravate the apoptosis induced by PRV and inhibit viral proliferation (Fig 4F).

### Circ29164 activates apoptosis by binding to ssc-miR-24-3p

**Fig 5:**
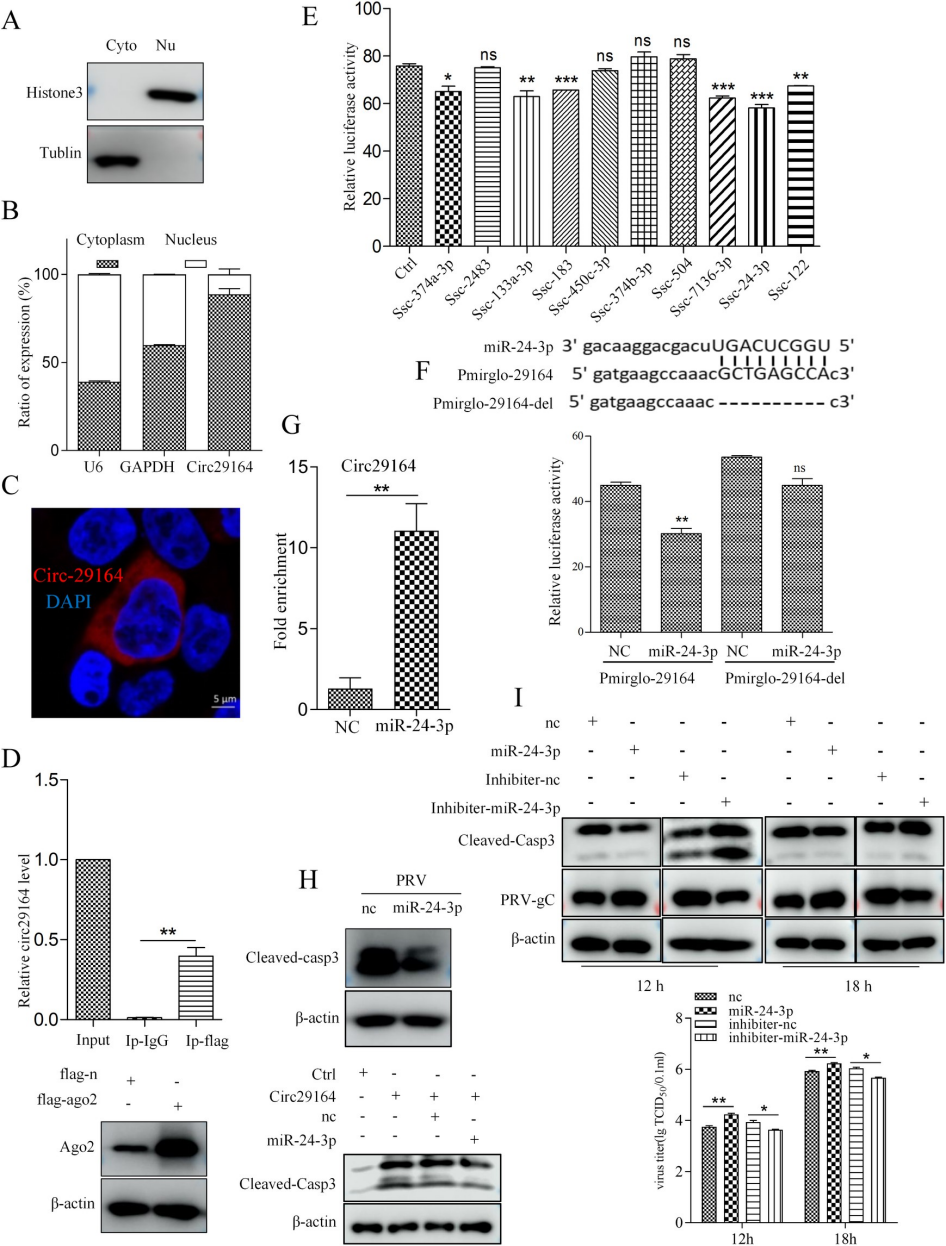
Circ29164 can sponge ssc-miR-24-3p. (A) PK-15 cells were fractionated into cytoplasmic (Cyto) and nuclear (Nu) fractions, and histone 3 and tubulin were detected to confirm the separation of Cyto and Nu. (B) The expression of circ29164 in the cytoplasm and nucleus as detected using RT-qPCR. (C) The subcellular localization of circ29164 as detected by RNA fluorescence *in situ* hybridization. (D) Circ29164 was enriched by AGO2 in an RNA immunoprecipitation assay. (E) Dual luciferase reporter assay for the luciferase activity of pmirglo-29164 in HEK-293 T cells transfected with 10 predicted miRNA mimics. (F) Identification of interaction site between circ29164 and ssc-miR-24-3p. 293T cells were cotransfected with ssc-miR-24-3p and pmirglo-29164 or pmirglo-29164-del, respectively, and luciferase activities were detected. (G) The interaction between ssc-miR-24-3p and circ29164. Biotinylated ssc-miR-24-3p was incubated with the lysate of cells overexpressing circ29164 and the enrichment of circ29164 was detected by RNA pull down assays. (H) ssc-miR-24-3p can repress the cell apoptosis induced by PRV-DX and circ29164. (I) The effect of ssc-miR-24-3p on PRV replication. Values are the means ± SDs. *, *p* < 0.05; **, *p* < 0.01; ***, *p* < 0.001.

Many circRNAs are reported to function as miRNA sponges. To investigate whether circ29164 acts as miRNA sponge, we determined the subcellular localization of circ29164. Nuclear and cytoplasmic fraction separation and fluorescence *in situ* hybridization showed that circ29164 was mainly located in the cytoplasm (Fig 5A–5C). A previous study showed that circRNAs in the cytoplasm bind to the AGO2 protein when they act as miRNA sponges [31]. Subsequently, we performed RNA immunoprecipitation for AGO2 in circ29164 overexpressing cells. RT-qPCR assays showed that circ29164 was specifically enriched by FLAG-AGO2 (Fig 5D), indicating that circ29164 might bind to miRNAs. Next, miRanda and psRobot were used to screen and predict potential circ29164-binding miRNAs. Ten miRNAs that might bind to circ29164 were selected as candidates. Subsequent dual luciferase reporter assays revealed that ssc-miR-24-3p significantly inhibited the luciferase activity of pmirglo-29164 (Fig 5E), indicating that circ29164 binds to ssc-miR-24-3p. To verify the interaction between circ29164 and ssc-miR-24-3p, the plasmid pmirglo-29164-del, which lacked the predicted miRNA binding site in circ29164, was constructed and cells cotransfected with the plasmid and ssc-miR-24-3p were subjected to dual luciferase reporter detection. The results showed ssc-miR-24-3p lost its ability to inhibit luciferase activity in cells transfected with pmirglo-29164-del (Fig 5F), indicating that the predicted motif ^46^GCTGAGCCA^54^ located in circ29164 is the binding site of ssc-miR-24-3p (UGGCUCAGU). To further confirm the binding between ssc-miR-24-3p and circ29164, a biotin-tagged ssc-miR-24-3p mimic was incubated with lysates from cells overexpressing circ29164. RNA pulldown assays showed that circ29164 was significantly enriched in the miR-24-3p-captured fraction compared with that in the negative control (Fig 5G). In contrast to circ29164 overexpression, miR-24-3p overexpression inhibited the cellular apoptosis induced by PRV infection and repressed the pro-apoptotic function of circ29164 (Fig 5H). Therefore, we assessed the influence of ssc-miR-24-3p on PRV proliferation. PK-15 cells were transfected with ssc-miR-24-3p or its inhibiter for 24 h and then infected with PRV of 0.5 MOI for indicated time. Detection of the viral protein and titer showed that ssc-miR-24-3p can inhibit apoptosis and promote viral proliferation (Fig 5I). The results indicated that circ29164 act as a competing endogenous RNA (ceRNA) to bind ssc-miR-24-3p and regulate viral proliferation.

### KEAP1 promotes apoptosis after circ29164 sponging of ssc-miR-24-3p

**Fig 6:**
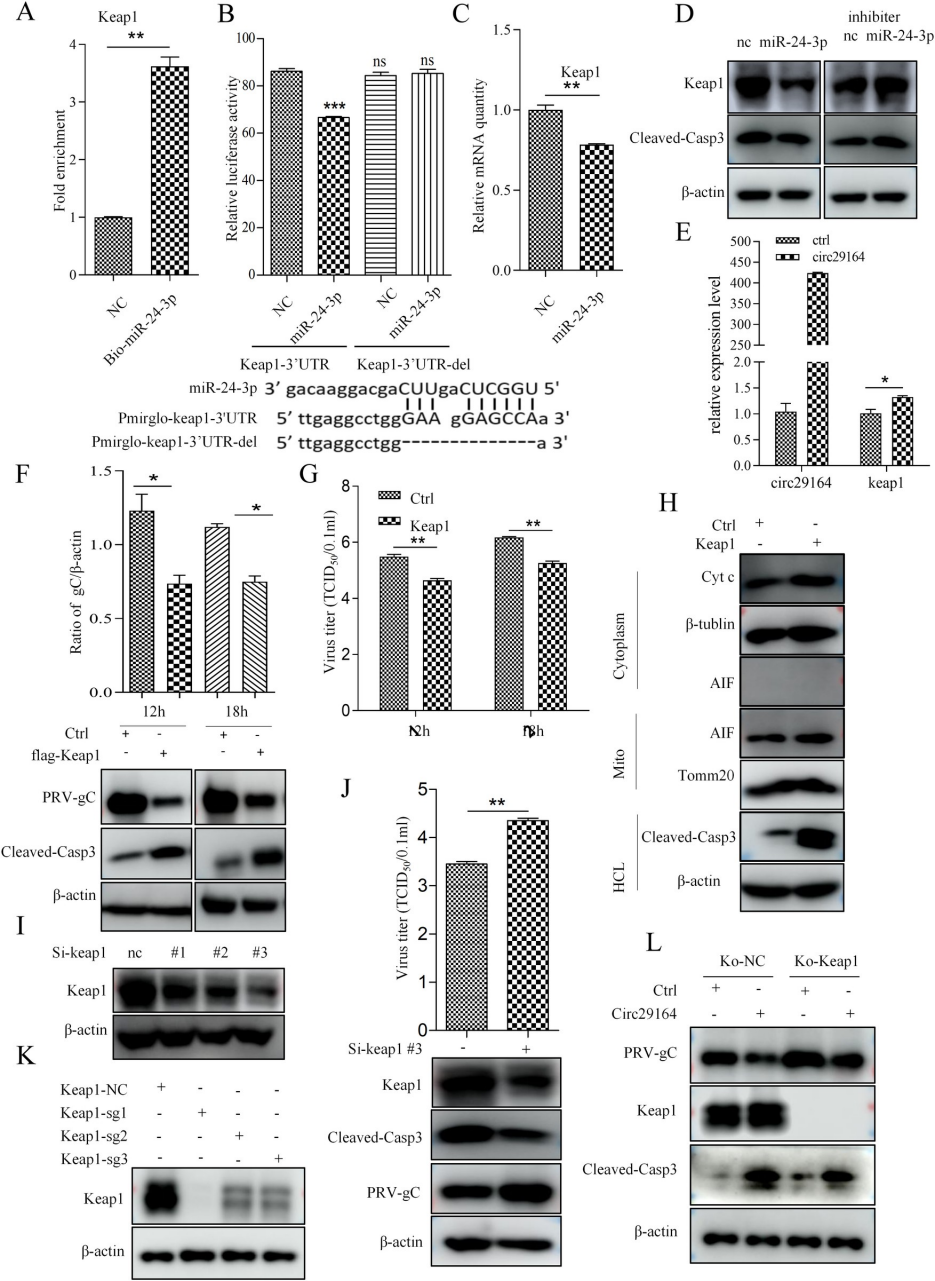
*Keap1* suppressed the replication of PRV by activating apoptosis as a target gene of ssc-miR-24-3p. (A) The interaction between ssc-miR-24-3p and *Keap1* as detected by RNA pull down assay. Biotin marked NC and ssc-miR-24-3p were incubated with the cell lysate. RNA was then enriched using streptavidin magnetic beads and the amount of *Keap1* RNA was quantified using RT-qPCR. (B) Identification of the interaction site between *Keap1* and ssc-miR-24-3p. ssc-miR-24-3p was co-transfected with pmirglo-keap1-3’UTR or pmirglo-keap1-3’UTR-del into 293T cells for 48 h, followed by detection of the luciferase activity. (C and D) The effect of ssc-miR-24-3p on the expression of *Keap1* mRNA and protein. ssc-miR-24-3p or the inhibitor were transfected into PK-15 cells for 36 h and the expression of *Keap1* mRNA and protein was detected using RT-qPCR and western blotting. (E) Circ29164 facilitates the expression of *Keap1*. pcDNA3.1-circ29164 was transfected into PK-15 cells for 36 h and *Keap1* mRNA expression was measured using RT-qPCR. (F and G) The effect of KEAP1 on cell apoptosis and the replication of PRV. PK-15 cells were induced to overexpress *Keap1* for 24 h and then infected by PRV-DX for the indicated times; subsequently, the viral protein level and the viral titer were measured. (H) Overexpression of *Keap1* induced the release of mitochondrial cytochrome C. (I and J) Interfering with *Keap1* expression inhibits cell apoptosis and promotes the replication of PRV. Si-keap1 was transfected into PK-15 cells for 36 h followed by the infection of PRV at the MOI of 0.5 for 18 h. (K) Screening of sgRNA for Keap1 knock out. (L) The effect of circ29164 on apoptosis and viral protein in Keap1 knockout cells. Values are the means ± SDs. *, *p* < 0.05; **, *p* < 0.01; ***, *p* < 0.001.

MicroRNAs regulate the function of target genes via recognition of cognate sequences in the 3′ untranslated region (UTR) of their mRNA. The target mRNAs bound by ssc-miR-24-3p were predicted using miRanda and psRobot. Combined with the previously reported effects of the predicted genes on apoptosis, *Prkch* (encoding protein kinase C eta), *Sox7* (encoding SRY-box transcription factor 7), and *Keap1* were selected for verification [32–35]. The biotin-marked synthetic ssc-miR-24-3p mimic was incubated with cell lysates to explore ssc-miR-24-3p binding with the target gene. RNA pull down assays showed that *Keap1* mRNA was significantly enriched by ssc-miR-24-3p compared with the control (Fig 6A), indicating ssc-miR-24-3p interacts with *Keap1* mRNA. Meanwhile, the wild-type 3′ UTR of *Keap1* (keap1-3′ UTR) and the predicted binding site deleted 3′ UTR of *Keap1* (keap1-3′ UTR-del) were constructed into pmir-GLO to detect their binding activity with ssc-miR-24-3p. The dual luciferase reporter assays indicated that ssc-miR-24-3p inhibited the luciferase expression from the wild-type *Keap1*-3′ UTR construct but not from the mutant construct (Fig 6B), indicating that the 3′ UTR of the *Keap1* mRNA is the target binding site of ssc-miR-24-3p (UGGCUCAGUUC, the same site as that in circ29164). Then, transfection of ssc-miR-24-3p into PK-15 cells showed inhibition of *Keap1* expression (Fig 6C–6D), whereas circ29164 promoted *Keap1* expression (Fig 6E).

*Keap1* is reported to involve in apoptosis [32]. To detect the role of *Keap1* interacted with ssc-miR-24-3p, the plasmid pCMV-flag-keap1 was constructed and transfected into PK-15 cells followed by the viral infection. The cell supernatants were collected for the detection of virus titer and the cell lysates were collected for immunoblotting detection. As expected, overexpressing of *Keap1* induced the release of cytoplasmic cyt *C* and cleaved-casp-3, and suppressed the replication of PRV (Fig 6F–6H). Furthermore, *Keap1* was knocked down by using small interfering RNAs (siRNAs) (Fig 6I). Subsequently, cell transfected by si-keap1#3 was used to detect apoptosis and PRV replication. The results showed that knockdown of *Keap1* inhibited the cell apoptosis and benefited to the viral replication (Fig 6J). Taken together, the results demonstrated that KEAP1 suppressed the replication of PRV by activating apoptosis. In summary, the aforementioned data suggest that circ29164 induces cell apoptosis by blocking ssc-miR-24-3p interaction with *Keap1*. To further assess the role of *Keap1* in the apoptosis induced by circ29164, the *Keap1* knock out (KO) PK15 cell line was screened from three sgRNA target sites (Fig 6K). *Keap1* KO PK15 cells were overexpressed with circ29164 by transfection for 24 h and then infected with PRV of 0.5 MOI for 12 h. Detection found that, despite some relief, circ29164 can still induce apoptosis and inhibits the replication of PRV (Fig 6L). The results suggested that, besides circ29164-miR-24-3p/KEAP1 axis, circ29164 may also regulate apoptosis and viral replication by other ways.

## Discussion

Noncoding RNAs, such as including miRNAs, long noncoding RNAs (lncRNAs), and circRNAs, were once regarded as junk transcripts from the genome. However, numerous studies, accompanied by high-throughput sequencing and bioinformatic analyses, have revealed that they can indeed be functional [36], especially lncRNAs and circRNAs. The functions of lncRNAs during virus infection have been reported, such as the regulation by lncRNAs of the replication of influenza A virus [37], herpes simplex virus-1 [38], swine influenza virus [39], porcine delta coronavirus [40], and PRV [41,42]. CircRNAs are novel noncoding RNA molecules with various biological functions and pathological implications [43,44]. Host cells might initiate a defense response after viral infection by altering circRNA expression [45,46]. In the present study, we identified an upregulated and RNase resistant noncoding RNA in virus-infected cells (Fig 1). This previously undiscovered circRNA, circ29164, has no protein encoding function, and participates in the cellular response to virus infection. In addition, circRNA and the parent mRNA were all from the alternative splicing of pre-mRNA. Hence, circRNA may change the expression of parent mRNA [47,48]. Here, we found that the infection of PRV significantly down-regulated the expression of EZH2 and that expression of EZH2 significantly promoted PRV proliferation. These results were consistent with the up-regulation of circ29164 and its inhibitory effect on PRV proliferation, indicating that host resist viral proliferation by down-regulation of mRNA and up-regulation of circ29164. However, the cellular factor or viral element that promotes circ29164 upregulation in virus-infected cells remains to be identified.

The “miRNA sponge” is the most conspicuous function of circRNAs [14,49]. Recent reports demonstrated that circRNA GATAD2A promotes H1N1 influenza A virus replication by inhibiting autophagy [46] and AIVR, a circRNA that absorbs an miRNA that degrades CREB binding protein (CREBBP) mRNA, antagonizes influenza virus by accelerating interferon beta production [50]. However, in the present study, circ29164 induced the release of cyt C from mitochondria and increased the level of cleaved caspase 3 by binding to ssc-miR-24-3p. Thereafter, *Keap1* was identified as the target mRNA of ssc-miR-24-3p, which binds to ssc-miR-24-3p at the same site as circ29164. KEAP1, a key factor of the KEAP1-nuclear factor erythroid 2-related factor 2 (NRF2) system, is an adaptor subunit of E3 ubiquitin ligase that regulates the activity of NRF2. NRF2 is a master regulator of cellular responses against environmental stresses and induces the expression of detoxification and antioxidant enzymes [51]. The KEAP1/NRF2 pathway is closely related to cellular oxidative stress and thus affects cell apoptosis [52]. Consequently, we concluded that circ29164 induces apoptosis by competitively binding to ssc-miR-24-3p, thereby relieving ssc-miR-24-3p-mediated inhibition of *Keap1* expression.

An intact intracellular environment is indispensable for virus proliferation; thus, apoptosis is a mechanism by which host cells resist virus invasion [53]. Initiating apoptosis programs is an effective way to limit the spread and proliferation of viruses [54]. Until now, the specific regulatory mechanism of apoptosis during PRV infection and the antiviral response of the host cells have remained unknown. In the present study, infection with PRV, PDCoV, and influenza virus subtypes caused the upregulation of circ29164. Moreover, *Keap1* overexpression induced apoptosis and suppressed virus replication. Therefore, we believe that circ29164 acts as an inhibitor of virus replication via KEAP1-induced apoptosis. Furthermore, considering that circ29164 is also likely to efficiently bind other miRNAs and that, despite some relief, circ29164 can still induce apoptosis and inhibits the replication of PRV in Keap1-Ko PK15 cells, circ29164 has multiple biological functions and may also regulate apoptosis and viral replication by other ways.

In summary, circ29164 sponges miR-24-3p, thereby indirectly promoting the expression of KEAP1, which results in cell apoptosis and the suppression of virus replication. These findings enriched our knowledge of the regulatory mechanisms of circRNAs during virus infection, and provide a deeper understanding concerning the use of apoptosis by the virus and the host cell.

## Materials and methods

### Cell and virus

Porcine kidney (PK-15) and ST (Swine Testis) cells, maintained in our laboratory, and human embryonic kidney HEK293T (293T) cells, purchased from the Chinese Academy of Sciences, were cultured at 37°C and 5% CO_2_ in Dulbecco’s modified Eagle’s medium (Gibco, CA, USA) containing 10% fetal bovine serum (FBS) (Biological Industries, Israel) and antibiotics (100 μg/ml streptomycin and 100U/ml penicillin).

The pseudorabies virus genotype II (PRV-DX), originally isolated from an infected pig, were stored in our laboratory and propagated in PK15 cells. Porcine delta coronavirus (PDCoV) strain CH-HA3-2017 was stored in our lab and propagated among ST cells. Influenza virus (IAV: H5N1, PR8) were stored in our lab and propagated in 9-day-old embryonated eggs at 37°C.

### Antibody and reagent

Mouse anti-flag mAb was purchased from Sigma-Aldrich (St. Louis, MO, USA); Rabbit anti-casp-3 (9665 and 9661) and rabbit anti-casp-8 (4790) mAbs were purchased from cell signaling technology; Mouse lgG (A7028), cell mitochondria isolation kit (C3601), annexin V-FITC apoptosis detection kit (C1062) and cell lysis buffer (P0013C and P0013F) were purchased from Beyotime (China); HRP-Goat Anti-Mouse, HRP-Goat Anti-Rabbit IgG were from KPL (Milford, MA, USA); Rabbit anti-KEAP1 mAb (10503-2-AP) was from Proteintech; Casp-9 colorimetric assay kits (APT139) was purchased from Sigma-Aldrich (St. Louis, MO, USA); Mouse anti-cytochrome C (ab110325) and rabbit anti-β-actin mAb (ab49846) were purchased from Abcam (Cambridge Science Park, United Kingdom).

### Construction of recombinant plasmids

The circ29164 overexpressing fragment (with flanking sequences), the flanking sequences (without the sequences of circ29164), the sequences of predicted ORFs and the sequences of *Keap1* were cloned into pcDNA3.1^+^ to generate 3.1-circ29164, 3.1-circ-flanking, predicted-ORF1, predicted-ORF2, predicted-ORF3, predicted-ORF4, linear-ROPN1-flag (constructed in previous study [42]), circ29164-FLAG and 3.1-flag-keap1. The gene of ago2 was cloned into pCMV-flag-n to generate Flag-ago2. The full length circ29164, circ29164-del, keap1-3UTR and keap1-3UTR-del were inserted into the pmirGLO plasmid (Promega).

### siRNAs, miRNA and transfection

Circ29164 (Si-29164#1: 5’-GAAGAGGAAACACCGAAGCTT-3’; Si-29164#2: 5’-CACCGAAGCAAAUUCUCGGTT-3’; Si-29164#3: 5’-AGGAAACACCGAAG CAAAUTT-3’) and *Keap1* siRNAs (Si-keap1#1: 5’-CCUGUCUUCAAGGCUAUGUTT-3’; Si-keap1#2: 5’-CCCGAGAGUA CAUCUACAUTT-3’; Si-keap1#3: 5’-GUCCUGCACAACUGUAUCUTT-3’), negative-control siRNAs, miRNA mimics were synthesized by GenePharma. For the transfection of plasmids, siRNA and miRNA, PK15 and 293T cells were cultured in the designated plates at a suitable density according to the experimental schemes and were transfected using jetPRIME (Polyplus Transfection, New York, NY, USA) according to the instruction.

### Viral infection and virus titration

Cells were infected with PRV-DX, PDCoV or IAV at the multiplicity of infection (MOI) of 0.5 and incubated in DMEM at 37°C for 1.5 h, then, replaced with DMEM containing 2% FBS after washed by phosphate buffer saline (PBS). PRV-DX titers in culture supernatants were determined by 50% tissue culture infectious dose (TCID_50_) by observing the cytopathic effect of vero cells in 96-well culture plate using Reed and Muench.

### RNase R resistance of circRNA

To detect the circRNA resistance to RNase R (Epicentre, USA), total RNA (6 μg) was digest by 20 units RNase R at 37°C for 30 min (the DEPC-treated water was used as control). The digested RNA was then purified by phenol-chloroform mixture and subjected to RT-qPCR to detect the level of circRNA.

### Real-time RT-qPCR analysis

Total RNAs were extracted and the concentrations were measured by using a spectrophotometer. Then, thermo scientific RevertAid First Strand cDNA Synthesis Kit (Thermo Fisher Scientific, USA) was used to synthesize cDNA from RNA. qPCR was performed in a total volume of 20 μl using AceQ qPCR SYBR Green Master Mix (Vazyme, China). Glyceraldehyde 3-phosphate dehydrogenase (GAPDH) was used as the internal control and the results were calculated with the ΔΔCt-method. Primers used in this study were listed in Table 1.

**Table 1:** Primers used in this study.

Primer name	Sequence (5’-3’)
3.1-circ29164-F	CGGGGTACCGGAAGAGGAAGAGAAGAAGGATGA
3.1-circ29164-R	CCCTCGAGACTCCAACAATCCAAAGCCTCTA
predicted-ORF1-R	CCCTCGAGCTACTTGTCGTCGTCGTCCTTGTAGTCGCCTGGGAACCTCCATATG
predicted-ORF2-R	CCCTCGAGTCACTTGTCGTCGTCGTCCTTGTAGTCGTGGGAAAAAAAAAGAAAAATAT
predicted-ORF3-R	CCCTCGAGCTACTTGTCGTCGTCGTCCTTGTAGTCGTCGGATTTGTTAACCACTGAGCC
predicted-ORF4-R	CCCTCGAGTCACTTGTCGTCGTCGTCCTTGTAGTCCAGGCAAATGCTCACGTGAT
circ29164-F	ACTGTGCCTCTTGTCAGGTGTATG
circ29164-R	TCGGGTGGCTCAGCGTTT
circ29164-F2	GAAGCAAATTCTCGGTGTCAAAC
circ29164-R2	CGGTGTTTCCTCTTCTTCTTTCT
circ29164-flag-F	CGGTTTTTTAGGACGACGACGACAAGGAAGCAAATTC
circ29164-flag-R	CTGACTCACCTTGTAGTCGGTGTTTCC
3.1-circ-flanking -F	AATTTCGGTTTTTTAGTGAGTCAGCAGGCA
3.1-circ-flanking -R	TGCCTGCTGACTCACTAAAAAACCGAAATT
flag-ago2 -F	CCGGAATTCATGTACTCGGGAGCCGGCC
flag-ago2 -R	AAATATGCGGCCGCTCACGCAAAGTACATGGTGCG
pmirglo-29164-F	CCGCTCGAGGAAGCAAATTCTCGGTGTCAAA
pmirglo-29164-R	ACGCGTCGACCGGTGTTTCCTCTTCTTCTTTCT
pmirglo-29164-del-F	AAAGATGAAGCCAAACACCCGAGAACGTGGAG
pmirglo-29164-del-R	CTCCACGTTCTCGGGTGTTTGGCTTCATCTTT
3.1-flag-keap1-F	CGGGGTACCATGGACTACAAGGACGACGATGACAAGATGCAACCGGAACCCAGGC
3.1-keap1-F	CGGGGTACCATGCAACCGGAACCCAGGC
3.1- keap1-R	ATAAGAATGCGGCCGCTCAACAGGTACAGTTCTGCTGGT
Keap1-sg1-F	CACCGGAAGTGCGAGATCCTGCAGT
Keap1-sg1-R	AAACACTGCAGGATCTCGCACTTCC
Keap1-sg2-F	CACCGGTACGCCTCCACGGAGTGCA
Keap1-sg2-R	AAACTGCACTCCGTGGAGGCGTACC
Keap1-sg3-F	CACCGGCTATGCGATGTTACGCTGC
Keap1-sg3-R	AAACGCAGCGTAACATCGCATAGCC
keap1 -F	CCCTGTCTTCAAGGCTATGTTCA
keap1 -R	GGAGCACACACTTCTCACCCAT
pmirglo-keap1-3UTR-F	CCGCTCGAGGCCACTTTTGTTTCTTGGGCA
pmirglo-keap1-3UTR- R	ACGCGTCGACTGGAAGACACTAGTTAGTTTGTTCT
pmirglo-keap1-3UTR-del-F	TGGGAAGGAGCCAAGCCCCTTCCTGCTTA
pmirglo-keap1-3UTR-del-R	TAAGCAGGAAGGGGCTTGGCTCCTTCCCA
EZH2-F	TTTCCAACACAAGTCATCCCGT
EZH2-R	TCAATAAAAGTCCCATCCTGGTCTA
circ29164 specific probe marked by cy3	GAGAATTTGCTTCGGTGTTTCCTCT

### Western blotting

Cells were washed with PBS and lysed in lysis buffer. Equal amounts of total protein were separated on SDS polyacrylamide gel electrophoresis and were then transferred onto nitrocellulose membranes (Merck Millipore), followed by blocking in PBS containing 5% skim milk for 1h. After washing with PBS containing 0.05% Tween 20 (PBST), the membrane was then incubated with primary antibodies overnight at 4°C and washed with PBST. Finally, the membranes were incubated with horseradish peroxidase–conjugated anti-mouse/rabbit IgG and were detected using enhanced chemiluminescence.

### Caspase assay

PK-15 cells, transfected with 3.1-circ-flanking and 3.1-circ29164 for 36 h, were collected and lysed. The cell lysates were then subjected to Western blotting, using a rabbit anti-casp-3 mAb and a rabbit anti-β-actin mAb. Casp-9 activities were detected by using casp-9 colorimetric assay kits according to the manufacturers’ instructions.

### TUNEL assay

The TUNEL assay kit (Beyotime Biotechnology, China, C1088) was used according to the instructions. First, PK15 cells were overexpressed with circ29164 for 36 h. Cells were fixed with 4% formaldehyde-PBS for 30 min, permeabilized with 0.5% Triton X-100 –PBS for 10 min. Normal cells incubated with 100 U DNase I (EN0521; Thermo) or not were used as a positive or negative control. Then, cells were incubated with terminal deoxynucleotidyltransferase (TdT) incubation buffer in the dark at 37°C for 1 h. After stained with DAPI at RT for 10 min, cells (green fluorescence) were observed under a fluorescence microscope.

### Cellular fractionation

The Paris kit (AM1921; Thermo) was used for the separation of nuclear and cytoplasmic components according to the instruction. Western blot analysis was performed with rabbit mAbs against histone H3 (R1105-1; Huaan Biological Technology) and beta-tubulin (0807–2; Huaan Biological Technology). In addition, the components of nuclear and cytoplasmic were subjected to RNA extraction and RT-qPCR to detect the distribution of circ29164.

### Detection of cyt c release from mitochondria

To isolate mitochondrial and cytosolic fractions, the cell mitochondria isolation kit was used according to the manufacturer’s instructions. Briefly, after the various treatments, cells were collected by centrifugation at 600 × *g* for 5 min at 4°C and then resuspended in 1 ml of 1× cytosol extraction buffer mix with added protease inhibitor cocktail and dithiothreitol (DTT; provided in the kit) before use. A pre-cold tissue homogenizer was then used to homogenize the cells. The homogenate was centrifuged at 800 × *g* for 10 min at 4°C, and the supernatant was subsequently centrifuged at 11,000 × *g* for 10 min at 4°C. The supernatant was transferred to a new tube as the cytosolic fraction, and the pellet was resuspended in 100 μl of mitochondrial extraction buffer as the mitochondrial fraction. Immunoblotting was performed with mouse mAbs to cyt *c* (ab110325; Abcam), β-actin (ab49846; Abcam), rabbit mAbs against AIF (5318; CST), and Tomm 20 (ab186734; Abcam).

### Immunoprecipitation

Take ago2 for example, flag-ago2 and 3.1-circ29164 were transfected into 293T cells for 48h. After washed by PBS, cells were treated with iced lysis buffer for 20 min. Centrifuging at 10,000 × *g* for 10 min at 4°C, the lysates were incubated with anti-flag or mouse lgG (as a control)for 5 h and then with Protein G beads (Beyotime, China) for 5 h. Then wash the beads 5 times and lysed into trizol reagent for RNA extraction and subjected to RT-qPCR for the detection of circ29164.

### Dual-luciferase assay

293T cells were seeded in 24-well plates and transfected with pmirglo-29164, pmirglo-29164-del, pmirglo-keap1-3UTR or pmirglo-keap1-3UTR-del together with a miRNA mimic or NC, then culture for another 48 h. Cells were collected and the luciferase activity was examined by Dual Luciferase Reporter Gene Assay Kit (Beyotime, China) according to the instruction using the multifunctional microplate reader. Relative luciferase activity (firefly luciferase/Renilla luciferase) was calculated and the change by the miRNA was obtained compared with NC.

### Biotin-coupled miRNA capture

Mock or circ29164 overexpressing PK-15 cells were harvested and lysed with lysis buffer supplemented with RNase and protease inhibitors for about 30 min. After centrifuging at 10000 × g for 10 min, 1/10 of the cell lysates were collected as input and the other cell lysates were incubated with 3’end biotinylated miR-24-3p mimic or control RNA (BioSune, China) for 6h at 4°C. Streptavidin-coupled magnetic beads (Life Technologies, USA) were then added and incubated for another 1.5h. Beads were washed for 5 times and lysed into trizol reagent. Then, RNA were extracted to detect the level of *Keap1* or circ29164 by using RT-qPCR.

### RNA FISH

Circ29164 specific probe marked by cy3 (table 1) was used for fluorescence *in situ* hybridization (FISH). In short, PK-15 cells were seeded into confocal dish and fixed with 4% paraformaldehyde (PFA) for 15 min and then incubated in ethanol for 12 h at 4°C, followed by permeabilization for 5 min in 0.5% Triton X-100. After briefly washed with wash buffer (Nuclease Free Water, 2x SSC and 10% formamide), cells were incubated with hybridization buffer (Nuclease Free Water, 2x SSC, 10% formamide and 100 mg/ml dextran sulfate sodium salt) containing FISH probe at 37°C for 12–15 h, and then washed again with wash buffer at 37°C for 30 min. Finally, nucleus was stained with DAPI and the cells were scanned with confocal microscope (Zeiss).

### Statistical analyses

The potential circ29164-binding miRNAs and the target mRNAs bound by ssc-miR-24-3p were predicted using miRanda and psRobot. GraphPad Prism (version 5.0) software was used for the data statistics and analysis. Quantitative data are presented by the mean ± standard deviation (SD) normalized to control and t-test was performed assuming equal variance (P < 0.05 was considered as statistically significant).

## Supporting information

S1 Supporting Information The original sequencing result of the junction of circ29164 in Fig 1B. (RAR)

S2 Supporting Information The original sequencing result of flag spanning junction of circ29164 in Fig 2D. (RAR)

S3 Supporting Information The original result of flow cytometry for detection of apoptosis in Fig 4D. (RAR)

S4 Supporting Information Minimal data set (values). (RAR)

S5 Supporting Information Raw_images. (RAR)

## References

[1] He W, Auclert LZ, Zhai X, Wong G, Zhang C, Zhu H, et al. Interspecies Transmission, Genetic Diversity, and Evolutionary Dynamics of Pseudorabies Virus. J INFECT DIS. 2019 2019-05-05;219(11):1705–15. doi: 10.1093/infdis/jiy731 30590733

[2] Mahjoub N, Dhorne-Pollet S, Fuchs W, Endale AM, Lange E, Klupp B, et al. A 2.5-kilobase deletion containing a cluster of nine microRNAs in the latency-associated-transcript locus of the pseudorabies virus affects the host response of porcine trigeminal ganglia during established latency. J VIROL. 2015 2015-01-01;89(1):428–42. doi: 10.1128/JVI.02181-14 25320324 PMC4301172

[3] Taharaguchi S, Kobayashi T, Yoshino S, Ono E. Analysis of regulatory functions for the region located upstream from the latency-associated transcript (LAT) promoter of pseudorabies virus in cultured cells. VET MICROBIOL. 2002 2002-03-22;85(3):197–208. doi: 10.1016/s0378-1135(01)00513-2 11852187

[4] Granstedt AE, Kuhn B, Wang SS, Enquist LW. Calcium imaging of neuronal circuits in vivo using a circuit-tracing pseudorabies virus. Cold Spring Harb Protoc. 2010 2010-04-01;2010(4):t5410. doi: 10.1101/pdb.prot5410 20360364 PMC3017426

[5] Boldogköi Z, Nógrádi A. Gene and cancer therapy—pseudorabies virus: a novel research and therapeutic tool? CURR GENE THER. 2003 2003-04-01;3(2):155–82. doi: 10.2174/1566523034578393 12653408

[6] Yang Y, Xu Z, Tao Q, Xu L, Gu S, Huang Y, et al. Construction of recombinant pseudorabies virus expressing PCV2 Cap, PCV3 Cap, and IL-4: investigation of their biological characteristics and immunogenicity. FRONT IMMUNOL. 2024 2024-01-20;15:1339387. doi: 10.3389/fimmu.2024.1339387 38571947 PMC10987767

[7] Sun YY, Liu KS, Zhang C, Ni Z, Zhu YC, Bao HL, et al. Recombinant pseudorabies virus (PRV) expressing stabilized E2 of classical swine fever virus (CSFV) protects against both PRV and CSFV. Antiviral Res. 2023 2023-03-01;211:105548. doi: 10.1016/j.antiviral.2023.105548 36702445

[8] Sun Y, Luo Y, Wang CH, Yuan J, Li N, Song K, et al. Control of swine pseudorabies in China: Opportunities and limitations. VET MICROBIOL. 2016 2016-02-01;183:119–24. doi: 10.1016/j.vetmic.2015.12.008 26790944

[9] Zhang H, Duan X, Liu G, Li Y, Dong S, Lin J, et al. Comparative transcriptomic analysis of PK15 cells infected with a PRV variant and the Bartha-K/61 vaccine strain. FRONT MICROBIOL. 2023 2023-01-20;14:1164170. doi: 10.3389/fmicb.2023.1164170 37213521 PMC10196252

[10] Li XD, Fu SH, Chen LY, Li F, Deng JH, Lu XC, et al. Detection of Pseudorabies Virus Antibodies in Human Encephalitis Cases. BIOMED ENVIRON SCI. 2020 2020-06-20;33(6):444–7. doi: 10.3967/bes2020.059 32641207

[11] Liu Q, Wang X, Xie C, Ding S, Yang H, Guo S, et al. A novel human acute encephalitis caused by pseudorabies virus variant strain. CLIN INFECT DIS. 2020 2020-07–15. 10.1093/cid/ciaa987 32667972

[12] Zhang XO, Dong R, Zhang Y, Zhang JL, Luo Z, Zhang J, et al. Diverse alternative back-splicing and alternative splicing landscape of circular RNAs. GENOME RES. 2016 2016-09-01;26(9):1277–87. doi: 10.1101/gr.202895.115 27365365 PMC5052039

[13] Zhang Y, Xue W, Li X, Zhang J, Chen S, Zhang JL, et al. The Biogenesis of Nascent Circular RNAs. CELL REP. 2016 2016-04-19;15(3):611–24. doi: 10.1016/j.celrep.2016.03.058 27068474

[14] Hansen TB, Jensen TI, Clausen BH, Bramsen JB, Finsen B, Damgaard CK, et al. Natural RNA circles function as efficient microRNA sponges. NATURE. 2013 2013-03-21;495(7441):384–8. doi: 10.1038/nature11993 23446346

[15] Conn VM, Hugouvieux V, Nayak A, Conos SA, Capovilla G, Cildir G, et al. A circRNA from SEPALLATA3 regulates splicing of its cognate mRNA through R-loop formation. NAT PLANTS. 2017 2017-04-18;3:17053. doi: 10.1038/nplants.2017.53 28418376

[16] Xue C, Li G, Zheng Q, Gu X, Bao Z, Lu J, et al. The functional roles of the circRNA/Wnt axis in cancer. MOL CANCER. 2022 2022-05-05;21(1):108. doi: 10.1186/s12943-022-01582-0 35513849 PMC9074313

[17] Qu L, Yi Z, Shen Y, Lin L, Chen F, Xu Y, et al. Circular RNA vaccines against SARS-CoV-2 and emerging variants. CELL. 2022 2022-05-12;185(10):1728–44. doi: 10.1016/j.cell.2022.03.044 35460644 PMC8971115

[18] Amaya L, Grigoryan L, Li Z, Lee A, Wender PA, Pulendran B, et al. Circular RNA vaccine induces potent T cell responses. Proc Natl Acad Sci U S A. 2023 2023-05-16;120(20):e1992776176. doi: 10.1073/pnas.2302191120 37155869 PMC10193964

[19] Liu CX, Li X, Nan F, Jiang S, Gao X, Guo SK, et al. Structure and Degradation of Circular RNAs Regulate PKR Activation in Innate Immunity. CELL. 2019 2019-05-02;177(4):865–80. doi: 10.1016/j.cell.2019.03.046 31031002

[20] Min J, Li Y, Li X, Wang M, Li H, Bi Y, et al. The circRNA circVAMP3 restricts influenza A virus replication by interfering with NP and NS1 proteins. PLOS PATHOG. 2023 2023-08-01;19(8):e1011577. doi: 10.1371/journal.ppat.1011577 37603540 PMC10441791

[21] Li H, Tang W, Jin Y, Dong W, Yan Y, Zhou J. Differential CircRNA Expression Profiles in PK-15 Cells Infected with Pseudorabies Virus Type II. VIROL SIN. 2020 2020-07-02. doi: 10.1007/s12250-020-00255-w 32617900 PMC7973350

[22] Galluzzi L, Maiuri MC, Vitale I, Zischka H, Castedo M, Zitvogel L, et al. Cell death modalities: classification and pathophysiological implications. CELL DEATH DIFFER. 2007 2007-07-01;14(7):1237–43. doi: 10.1038/sj.cdd.4402148 17431418

[23] Kroemer G, Galluzzi L, Brenner C. Mitochondrial membrane permeabilization in cell death. PHYSIOL REV. 2007 2007-01-01;87(1):99–163. doi: 10.1152/physrev.00013.2006 17237344

[24] Modjtahedi N, Giordanetto F, Madeo F, Kroemer G. Apoptosis-inducing factor: vital and lethal. TRENDS CELL BIOL. 2006 2006-05-01;16(5):264–72. doi: 10.1016/j.tcb.2006.03.008 16621561

[25] Li LY, Luo X, Wang X. Endonuclease G is an apoptotic DNase when released from mitochondria. NATURE. 2001 2001-07-05;412(6842):95–9. doi: 10.1038/35083620 11452314

[26] Liu X, Kim CN, Yang J, Jemmerson R, Wang X. Induction of apoptotic program in cell-free extracts: requirement for dATP and cytochrome c. CELL. 1996 1996-07-12;86(1):147–57. doi: 10.1016/s0092-8674(00)80085-9 8689682

[27] Lorenzo HK, Susin SA, Penninger J, Kroemer G. Apoptosis inducing factor (AIF): a phylogenetically old, caspase-independent effector of cell death. CELL DEATH DIFFER. 1999 1999-06-01;6(6):516–24. doi: 10.1038/sj.cdd.4400527 10381654

[28] Acehan D, Jiang X, Morgan DG, Heuser JE, Wang X, Akey CW. Three-dimensional structure of the apoptosome: implications for assembly, procaspase-9 binding, and activation. MOL CELL. 2002 2002-02-01;9(2):423–32. doi: 10.1016/s1097-2765(02)00442-2 11864614

[29] Yang Y, Gao X, Zhang M, Yan S, Sun C, Xiao F, et al. Novel Role of FBXW7 Circular RNA in Repressing Glioma Tumorigenesis. J Natl Cancer Inst. 2018 2018-03-01;110(3):304–15. doi: 10.1093/jnci/djx166 28903484 PMC6019044

[30] Galluzzi L, Vitale I, Aaronson SA, Abrams JM, Adam D, Agostinis P, et al. Molecular mechanisms of cell death: recommendations of the Nomenclature Committee on Cell Death 2018. CELL DEATH DIFFER. 2018 2018-03-01;25(3):486–541. doi: 10.1038/s41418-017-0012-4 29362479 PMC5864239

[31] Qu S, Yang X, Li X, Wang J, Gao Y, Shang R, et al. Circular RNA: A new star of noncoding RNAs. CANCER LETT. 2015 2015-09-01;365(2):141–8. doi: 10.1016/j.canlet.2015.06.003 26052092

[32] Xiao X, Lu Z, Lin V, May A, Shaw DH, Wang Z, et al. MicroRNA miR-24-3p Reduces Apoptosis and Regulates Keap1-Nrf2 Pathway in Mouse Cardiomyocytes Responding to Ischemia/Reperfusion Injury. OXID MED CELL LONGEV. 2018 2018-01-20;2018:7042105. doi: 10.1155/2018/7042105 30622671 PMC6304907

[33] Yan L, Ma J, Zhu Y, Zan J, Wang Z, Ling L, et al. miR-24-3p promotes cell migration and proliferation in lung cancer by targeting SOX7. J CELL BIOCHEM. 2018 2018-05-01;119(5):3989–98. doi: 10.1002/jcb.26553 29231262

[34] Zhang MX, Zhang J, Zhang H, Tang H. miR-24-3p Suppresses Malignant Behavior of Lacrimal Adenoid Cystic Carcinoma by Targeting PRKCH to Regulate p53/p21 Pathway. PLOS ONE. 2016 2016-01-20;11(6):e158433. doi: 10.1371/journal.pone.0158433 27351203 PMC4924841

[35] Li X, Xu L, Hou X, Geng J, Tian J, Liu X, et al. Advanced Oxidation Protein Products Aggravate Tubulointerstitial Fibrosis Through Protein Kinase C-Dependent Mitochondrial Injury in Early Diabetic Nephropathy. Antioxid Redox Signal. 2019 2019-03-20;30(9):1162–85. doi: 10.1089/ars.2017.7208 29482336

[36] Adelman K, Egan E. Non-coding RNA: More uses for genomic junk. NATURE. 2017 2017-03-08;543(7644):183–5. doi: 10.1038/543183a 28277509

[37] Imamura K, Imamachi N, Akizuki G, Kumakura M, Kawaguchi A, Nagata K, et al. Long noncoding RNA NEAT1-dependent SFPQ relocation from promoter region to paraspeckle mediates IL8 expression upon immune stimuli. MOL CELL. 2014 2014-02-06;53(3):393–406. doi: 10.1016/j.molcel.2014.01.009 24507715

[38] Wang Z, Fan P, Zhao Y, Zhang S, Lu J, Xie W, et al. NEAT1 modulates herpes simplex virus-1 replication by regulating viral gene transcription. CELL MOL LIFE SCI. 2017 2017-03-01;74(6):1117–31. doi: 10.1007/s00018-016-2398-4 27783096 PMC5309293

[39] Zhang Y, Yu T, Ding Y, Li Y, Lei J, Hu B, et al. Analysis of Expression Profiles of Long Noncoding RNAs and mRNAs in A549 Cells Infected with H3N2 Swine Influenza Virus by RNA Sequencing. VIROL SIN. 2020 2020-04-01;35(2):171–80. doi: 10.1007/s12250-019-00170-9 31777011 PMC7198687

[40] Liu J, Wang F, Du L, Li J, Yu T, Jin Y, et al. Comprehensive Genomic Characterization Analysis of lncRNAs in Cells With Porcine Delta Coronavirus Infection. FRONT MICROBIOL. 2019 2019-01-20;10:3036. doi: 10.3389/fmicb.2019.03036 32063887 PMC6999024

[41] Fang L, Gao Y, Liu X, Bai J, Jiang P, Wang X. Long non-coding RNA LNC_000641 regulates pseudorabies virus replication. VET RES. 2021 2021-03-25;52(1):52. doi: 10.1186/s13567-021-00922-0 33766129 PMC7992786

[42] Jin Y, Zhang K, Huang W, Tang W, Li H, Dong W, et al. Identification of functional lncRNAs in pseudorabies virus type II infected cells. VET MICROBIOL. 2020 2020-03-01;242:108564. doi: 10.1016/j.vetmic.2019.108564 32122616

[43] Du WW, Yang W, Liu E, Yang Z, Dhaliwal P, Yang BB. Foxo3 circular RNA retards cell cycle progression via forming ternary complexes with p21 and CDK2. NUCLEIC ACIDS RES. 2016 2016-04-07;44(6):2846–58. doi: 10.1093/nar/gkw027 26861625 PMC4824104

[44] Rybak-Wolf A, Stottmeister C, Glažar P, Jens M, Pino N, Giusti S, et al. Circular RNAs in the Mammalian Brain Are Highly Abundant, Conserved, and Dynamically Expressed. MOL CELL. 2015 2015-06-04;58(5):870–85. doi: 10.1016/j.molcel.2015.03.027 25921068

[45] Li H, Tang W, Jin Y, Dong W, Yan Y, Zhou J. Differential CircRNA Expression Profiles in PK-15 Cells Infected with Pseudorabies Virus Type II. VIROL SIN. 2021 2021-02-01;36(1):75–84. doi: 10.1007/s12250-020-00255-w 32617900 PMC7973350

[46] Yu T, Ding Y, Zhang Y, Liu Y, Li Y, Lei J, et al. Circular RNA GATAD2A promotes H1N1 replication through inhibiting autophagy. VET MICROBIOL. 2019 2019-04-01;231:238–45. doi: 10.1016/j.vetmic.2019.03.012 30955816

[47] Abdelmohsen K, Panda AC, Munk R, Grammatikakis I, Dudekula DB, De S, et al. Identification of HuR target circular RNAs uncovers suppression of PABPN1 translation by CircPABPN1. RNA BIOL. 2017 2017-03-04;14(3):361–9. doi: 10.1080/15476286.2017.1279788 28080204 PMC5367248

[48] Ou R, Mo L, Tang H, Leng S, Zhu H, Zhao L, et al. circRNA-AKT1 Sequesters miR-942-5p to Upregulate AKT1 and Promote Cervical Cancer Progression. Mol Ther Nucleic Acids. 2020 2020-06-05;20:308–22. doi: 10.1016/j.omtn.2020.01.003 32193155 PMC7078494

[49] Zheng Q, Bao C, Guo W, Li S, Chen J, Chen B, et al. Circular RNA profiling reveals an abundant circHIPK3 that regulates cell growth by sponging multiple miRNAs. NAT COMMUN. 2016 2016-04-06;7:11215. doi: 10.1038/ncomms11215 27050392 PMC4823868

[50] Qu Z, Meng F, Shi J, Deng G, Zeng X, Ge J, et al. A Novel Intronic Circular RNA Antagonizes Influenza Virus by Absorbing a microRNA That Degrades CREBBP and Accelerating IFN-β Production. MBIO. 2021 2021-08-31;12(4):e101721. 10.1128/mBio.01017-21 PMC8406138 34281396

[51] Suzuki T, Yamamoto M. Molecular basis of the Keap1-Nrf2 system. Free Radic Biol Med. 2015 2015-11-01;88(Pt B):93–100. doi: 10.1016/j.freeradbiomed.2015.06.006 26117331

[52] Wang J, Ishfaq M, Xu L, Xia C, Chen C, Li J. METTL3/m(6)A/miRNA-873-5p Attenuated Oxidative Stress and Apoptosis in Colistin-Induced Kidney Injury by Modulating Keap1/Nrf2 Pathway. FRONT PHARMACOL. 2019 2019-01-20;10:517. doi: 10.3389/fphar.2019.00517 31156435 PMC6530351

[53] Zhou X, Jiang W, Liu Z, Liu S, Liang X. Virus Infection and Death Receptor-Mediated Apoptosis. Viruses. 2017 2017-10-27;9(11). doi: 10.3390/v9110316 29077026 PMC5707523

[54] Kvansakul M. Viral Infection and Apoptosis. Viruses. 2017 2017-11-23;9(12). doi: 10.3390/v9120356 29168732 PMC5744131

